# Identification of Common Molecular Signatures in Chronic Obstructive Pulmonary Disease and Pulmonary Tuberculosis

**DOI:** 10.3390/cimb48050462

**Published:** 2026-04-29

**Authors:** Stanislav Kotlyarov, Dmitry Oskin

**Affiliations:** 1 Department of Nursing, Ryazan State Medical University, 390026 Ryazan, Russia; 2 Department of Infectious Diseases and Phthisiology, Ryazan State Medical University, 390026 Ryazan, Russia; doctor.oskin@yandex.ru

**Keywords:** COPD, tuberculosis, pathogenetic mechanisms, bioinformatics analysis, differentially expressed genes, hub genes

## Abstract

Chronic obstructive pulmonary disease (COPD) and pulmonary tuberculosis (TB) are major causes of morbidity and mortality worldwide. Epidemiologic studies indicate an increased risk of tuberculosis in patients with COPD; however, the shared molecular mechanisms underlying the pathogenesis of these two diseases remain insufficiently understood. Objective. Based on a comparative bioinformatics analysis of peripheral blood transcriptomic profiles in patients with COPD and pulmonary tuberculosis, to identify common systemic immune mechanisms associated with the pathogenesis of both diseases. Gene expression data from the NCBI GEO public database were analyzed. GSE34608 included blood samples from 8 patients with tuberculosis and 18 healthy controls. The GSE76705 dataset contained peripheral-blood samples from 364 former smokers (225 with COPD and 139 without). Functional enrichment (GO Biological Process and KEGG) was run in ShinyGO; protein–protein interaction networks were built in STRING, and the top-15 hub genes were ranked by the MCC algorithm in CytoHubba. In tuberculosis, 892 up-regulated and 1448 down-regulated genes were identified; in COPD, 520 up-regulated and 1329 down-regulated. Common upregulated DEGs are involved in toll-like receptor signaling pathways, NOD-like receptor signaling pathways, neutrophil extracellular trap (NET) formation, phagosomes, and tuberculosis. Downregulated genes in each of the diseases were associated with processes of transcriptional regulation and RNA metabolism, which may indicate common transcriptional abnormalities in COPD and tuberculosis. COPD and tuberculosis share common pathogenic mechanisms, including the activation of innate immune signaling pathways (TLR, NOD), neutrophilic inflammation, the formation of neutrophil extracellular traps (NETosis), and phagocyte dysfunction. The identified common genes and signaling pathways may serve as a basis for the development of biomarkers and therapeutic targets; however, they require further validation in independent cohorts.

## 1. Introduction

Chronic obstructive pulmonary disease (COPD) and pulmonary tuberculosis are major socioeconomic public health problems and are leading contributors to morbidity and mortality from respiratory diseases worldwide [1,2,3,4]. According to the World Health Organization, COPD is the third leading cause of death globally, accounting for approximately 3.23 million deaths annually [5,6]. Tuberculosis, an infectious disease, also remains among the 10 leading causes of death, with approximately 10.6 million new cases reported in 2022 [7]. Despite their different etiologies, these two diseases frequently coexist in the same patient, resulting in mutual aggravation of the clinical course and a worse prognosis [8,9,10,11,12].

COPD is characterized by chronic airway inflammation, remodeling of the bronchial wall, and the development of emphysema, ultimately leading to irreversible airflow limitation [13,14,15]. Its principal etiologic factor is prolonged exposure to inhaled noxious agents, primarily tobacco smoke [16]. In contrast, pulmonary tuberculosis is caused by *Mycobacterium tuberculosis* and is characterized by granulomatous inflammation, caseous necrosis, and fibrosis of lung tissue [17,18]. Despite these differences, epidemiologic studies convincingly demonstrate that COPD is an independent risk factor for tuberculosis [9,19]. Patients with COPD have a 2- to 3-fold higher risk of developing tuberculosis, which is associated with impaired mucociliary clearance and local and systemic immune disturbances [20,21].

In recent decades, increasing evidence has suggested that common pathogenetic mechanisms underlie the comorbidity of COPD and tuberculosis [22]. Chronic inflammation, innate immune dysfunction, and impaired tissue repair play key roles in the development of both diseases [20,23,24]. In particular, macrophages, which are central cells of innate immunity, exhibit an alternatively activated phenotype and impaired phagocytic activity in COPD, whereas in tuberculosis they serve as the primary niche for mycobacterial persistence [25,26,27]. However, the molecular mechanisms determining the commonality of pathogenesis in these two diseases remain insufficiently elucidated.

Modern bioinformatics approaches to transcriptomic data analysis provide new opportunities to identify shared pathogenetic signatures [28,29]. Analysis of differentially expressed genes (DEGs), enrichment of biological processes and signaling pathways, and construction of protein–protein interaction (PPI) networks make it possible to identify key regulatory molecules integrating pathological processes across diseases [28,30,31,32].

Aim of the study: To identify common systemic immune mechanisms associated with the pathogenesis of both COPD and pulmonary tuberculosis based on a comparative bioinformatics analysis of peripheral blood transcriptomic profiles in patients with these conditions, followed by functional annotation of differentially expressed genes and analysis of protein–protein interactions.

The novelty of the current study lies in the comparative transcriptomic analysis using peripheral blood datasets, which allows for the identification of systemic immune mechanisms common to both diseases. Unlike previous studies, we analyzed upregulated and downregulated genes separately, which allowed us to identify not only activated but also suppressed pathological pathways.

## 2. Materials and Methods

### 2.1. Data Collection

The analysis was performed using datasets obtained from the National Center for Biotechnology Information (NCBI) Gene Expression Omnibus (GEO). Gene expression data from datasets GSE34608 and GSE76705 were used.

From GSE34608, gene expression data from blood samples of 8 patients with pulmonary tuberculosis and 18 healthy controls were included in the analysis. The data were generated using the GPL17077 Agilent-039494 SurePrint G3 Human GE v2 8 × 60K Microarray 039381 platform (probe name version). Data normalization was performed using a locally weighted scatterplot smoothing (LOWESS) analysis [33].

The GSE76705 dataset included gene expression profiles in peripheral blood from 364 former smokers from two cohorts: ECLIPSE and COPDGene [34,35]. A comparison was made between a group of patients with COPD (FEV1/FVC < 0.7; *n* = 225) and a control group of former smokers without COPD (FEV1/FVC ≥ 0.7; *n* = 139). Expression profiling was performed using the Affymetrix Human Genome U133 Plus 2.0 Array (GPL570) platform. Pre-processed and normalized data provided by the study authors were used for analysis. Normalization was performed using the Robust Multi-array Average (RMA) method, followed by quantile normalization.

### 2.2. Differential Expression Analysis

To assess differentially expressed genes, a bioinformatics analysis was performed in the comparison groups using the limma package in R (v. 4.0.2) and the Phantasus application (v. 1.11.0) [36]. Differential expression analysis was performed independently for each dataset. We conducted two independent differential expression analyses and then compared the gene lists, requiring that the direction of change be consistent. This conservative approach minimizes artifacts associated with platform differences. The Benjamini–Hochberg algorithm was used to adjust the level of statistical significance for multiple comparisons (false discovery rate, FDR). The criteria for screening differentially expressed genes in the GSE34608 dataset were an absolute |logFC| value > 1 and *p*-values satisfying the condition FDR ≤ 5%. The criteria for screening differentially expressed genes in the GSE76705 dataset were an absolute |logFC| value > 0.2 and *p*-values satisfying the condition FDR ≤ 5%. The threshold of |logFC| > 0.2 for the COPD dataset was selected to balance the number of identifiable differentially expressed genes, their biological interpretability, and statistical rigor, taking into account the smaller effect sizes typical of peripheral blood transcriptomic changes in chronic, non-infectious inflammatory conditions compared with acute infectious diseases. Common DEGs were identified using the FunRich tool (v. 3.1.3) with Venn diagram construction. Genes were considered shared when they changed in the same direction across all datasets (upregulated in all datasets or downregulated in all datasets).

### 2.3. Gene Ontology Analysis

Gene ontology analysis was performed to identify specific biological and molecular pathways using ShinyGO v0.85, according to biological processes from THE GENE ONTOLOGY RESOURCE consortium database, as well as pathway identification based on the Kyoto Encyclopedia of Genes and Genomes (KEGG) and the Reactome database. A Benjamini–Hochberg-adjusted *p* value < 0.05 was used as the threshold for identification of biological processes and pathways.

The molecular protein–protein interactions (PPI) among the protein products of common DEGs were assessed using the Search Tool for the Retrieval of Interacting Genes (STRING) online database. An interaction score of at least 0.4 (medium confidence) was considered significant. PPIs were visualized using Cytoscape v.3.10.3. Relationships among DEGs were analyzed using the Network Analyzer plugin in Cytoscape. In addition, Molecular Complex Detection (MCODE) was used to identify gene clusters in the PPI network. The cutoff criteria were set as follows: degree cutoff = 2, node score cutoff = 0.2, k-core = 2, and max depth = 100.

In addition, the most important genes in the network were identified using the cytoHubba application in Cytoscape. The Cytoscape cytoHubba plugin was used to rank network nodes according to their network characteristics. The Maximal Clique Centrality (MCC) topological analysis algorithm was used for analysis, prediction, and visualization of key proteins in the molecular PPI network. The MCC algorithm was chosen because it is one of the most reliable methods for ranking nodes in PPI networks, enabling the identification of the most central and functionally significant genes. The selection of the top 15 genes is based on a balance between statistical power and biological interpretability.

## 3. Results

### 3.1. Identification of Differentially Expressed Genes

The bioinformatics analysis revealed statistically significant differences in gene expression between the comparison groups. In dataset GSE34608, using the selected cutoff criteria, 892 upregulated DEGs and 1448 downregulated DEGs were identified. Using the selected cutoff criteria, 520 differentially expressed genes with upregulation and 1329 differentially expressed genes with downregulation were identified in the GSE76705 dataset.

### 3.2. Enrichment Analysis of Differentially Expressed Genes

#### 3.2.1. Enrichment Analysis of Differentially Expressed Genes in Tuberculosis

**Figure 1:**
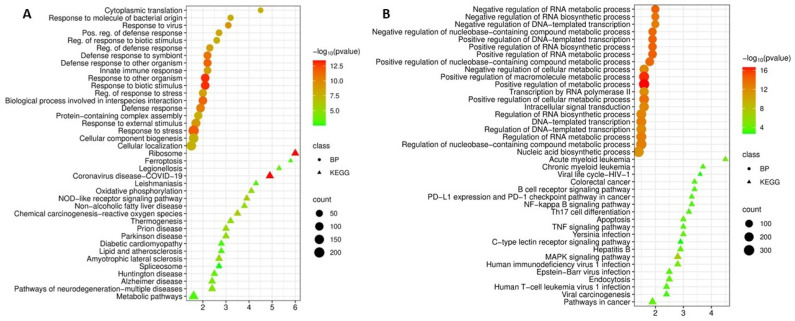
(**A**) Functional enrichment analysis of biological processes (BP) and KEGG pathways of upregulated DEGs in tuberculosis versus control; (**B**) Functional enrichment analysis of biological processes and KEGG pathways of downregulated DEGs in tuberculosis versus control.

Enrichment analysis of upregulated DEGs using the Gene Ontology Biological Process database revealed their predominant involvement in immune and host defense responses. The most significantly enriched processes were response to other organisms (*p* = 9.6 × 10^−14^), defense response (*p* = 3.4 × 10^−11^), innate immune response (*p* = 2.2 × 10^−9^), and response to stress (*p* = 1.6 × 10^−12^). Notably, there was marked representation of processes associated with response to molecules of bacterial origin (*p* = 9.4 × 10^−9^) and response to viruses (*p* = 4.2 × 10^−10^), as well as regulation of defense response (*p* = 2.9 × 10^−9^). Among the most significant processes, cytoplasmic translation was also identified (enrichment 4.5, *p* = 1.3 × 10^−8^), which may indicate high metabolic activity of the cells (Figure 1A).

KEGG pathway analysis of upregulated genes showed significant enrichment in metabolic pathways (*p* = 8.6 × 10^−4^). The highest enrichment scores were observed for ribosomes (enrichment 6, *p* = 3.7 × 10^−14^), ferroptosis (enrichment 5.8, *p* = 1.0 × 10^−3^), and infectious disease pathways such as legionellosis and leishmaniasis. The key role of innate immunity in tuberculosis pathogenesis was further supported by enrichment of the NOD-like receptor signaling pathway (*p* = 1.2 × 10^−6^) as well as pathways related to oxidative phosphorylation (*p* = 8.6 × 10^−6^) and chemical carcinogenesis-reactive oxygen species (*p* = 1.2 × 10^−6^) (Figure 1A).

In contrast to upregulated genes, enrichment analysis of downregulated DEGs revealed their key role in transcriptional regulation. The most significantly enriched biological processes were positive and negative regulation of transcription, including DNA-templated transcription (*p* = 4.4 × 10^−15^ and *p* = 1.6 × 10^−13^, respectively), and regulation of RNA metabolic processes (*p* = 1.6 × 10^−13^). The data obtained may indicate a profound restructuring of cellular transcriptional activity in patients with tuberculosis, affecting both the activation and repression of a wide range of genes. Intracellular signal transduction (*p* = 6.4 × 10^−13^) and transcription by RNA polymerase II (*p* = 1.6 × 10^−12^) were also identified among the enriched processes, which may highlight the complexity of regulatory changes in response to infection (Figure 1B).

KEGG pathway enrichment analysis of downregulated genes demonstrated their involvement in oncogenesis and signaling pathways critical for immune cell function. The greatest enrichment was observed for acute myeloid leukemia (enrichment 4.5, *p* = 3.6 × 10^−5^), chronic myeloid leukemia (enrichment 3.7, *p* = 2.8 × 10^−4^), and colorectal cancer (enrichment 3.4, *p* = 3.2 × 10^−4^). The identified enrichment of the B-cell receptor signaling pathway, PD-L1 expression, PD-1 checkpoint pathway, NF-kappa B signaling pathway, and Th17 cell differentiation may indicate the suppression of key immunoregulatory mechanisms. The most statistically significant enrichment was observed for the MAPK signaling pathway (*p* = 1.1 × 10^−7^), which plays a central role in cell proliferation, differentiation, and apoptosis. In addition, decreased expression affected genes involved in apoptosis (*p* = 1.6 × 10^−4^) and responses to viral infections (Epstein–Barr virus, HIV-1, and hepatitis B), which may reflect impaired elimination of infected cells and antiviral defense (Figure 1B).

#### 3.2.2. Functional Analysis of Key Hub Genes in Tuberculosis

To validate the results of the genome-wide analysis and identify central regulators of the pathological process, enrichment analysis was performed for the 15 most significant upregulated genes identified by Cytoscape (*PTGS2*, *CASP1*, *FCGR2A*, *TLR4*, *CCR2*, *JAK2*, *TLR5*, *TLR7*, *FCGR3B*, *IL1B*, *TNFSF13B*, *CD274*, *IL15*, *ARG1*, *CD163*).

GO Biological Process analysis of this gene set confirmed its key role in the development of inflammatory and immune responses. The highest enrichment scores were observed for cellular response to mechanical stimulus and cellular response to external stimulus (enrichment ~122–124), which may reflect the response of immune cells to microenvironmental changes during infection. Significant enrichment was also identified for positive regulation of inflammatory response (enrichment 58.7, *p* = 1.0 × 10^−9^) and positive regulation of cytokine production (enrichment 26.5, *p* = 4.3 × 10^−12^).

**Figure 2:**
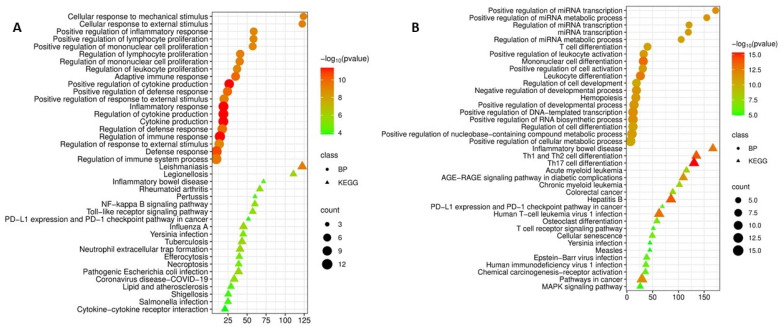
(**A**) Functional enrichment analysis of biological processes (BP) and KEGG pathways of the top 15 most significant upregulated differentially expressed genes in tuberculosis versus control; (**B**) Functional enrichment analysis of biological processes and KEGG pathways of the top 15 most significant downregulated differentially expressed genes in tuberculosis versus control.

Moreover, the identified genes were closely associated with adaptive immune responses, including positive regulation of lymphocyte proliferation (enrichment 58.4, *p* = 1.0 × 10^−9^) and adaptive immune response based on somatic recombination of immune receptors (enrichment 34.9, *p* = 1.2 × 10^−10^). These findings may indicate that the identified hub genes coordinate both innate and adaptive immune mechanisms in tuberculosis infection (Figure 2A).

An enrichment analysis based on the KEGG database revealed that the proteins encoded by these genes are involved in the pathogenesis of a wide range of infectious and autoimmune diseases, including enrichment in the tuberculosis signaling pathway (enrichment 43.3, *p* = 1.7 × 10^−6^), which may confirm the relevance of the selected genes to the disease under study.

In the analysis of biological processes, the identified genes were significantly associated with key innate immunity pathways: the NF-kappa B signaling pathway (enrichment 60.2, *p* = 4.9 × 10^−6^) and the toll-like receptor signaling pathway (enrichment 57.4, *p* = 5.3 × 10^−6^), as well as programmed cell death processes such as necroptosis (enrichment 39, *p* = 1.9 × 10^−5^) and efferocytosis (enrichment 39.8, *p* = 1.9 × 10^−5^). In addition, enrichment of the PD-L1 expression and PD-1 checkpoint pathway (enrichment 51.7, *p* = 1.2 × 10^−4^) was identified, which may indicate involvement of immune exhaustion mechanisms in tuberculosis pathogenesis (Figure 2A).

Additional enrichment analysis was performed for the 15 most significant downregulated genes identified in tuberculosis (*TGFB1*, *SPI1*, *CD4*, *STAT6*, *STAT5B*, *JUN*, *RELA*, *RUNX3*, *SMAD3*, *RUNX1*, *GATA3*, *BCL2*, *CEBPA*, *MYC*, *NFATC1*). The results revealed fundamentally different biological processes associated with this group of genes, which may indicate the complexity of transcriptional reprogramming during infection.

GO Biological Process analysis demonstrated exceptionally high enrichment for processes associated with microRNA regulation. The most significant were positive regulation of miRNA transcription (enrichment 172.3, *p* = 2.1 × 10^−12^) and regulation of miRNA metabolic processes (enrichment 105.4, *p* = 2.0 × 10^−11^). This may indicate a potential role for down-regulated genes in controlling post-transcriptional regulation of gene expression in tuberculosis.

In addition, functional enrichment analysis of downregulated genes revealed their involvement in the differentiation and development of immune cells. Significant enrichment was observed for T cell differentiation (enrichment 40.1, *p* = 2.0 × 10^−11^), mononuclear cell differentiation (enrichment 31.9, *p* = 5.0 × 10^−14^), hemopoiesis (enrichment 17.4, *p* = 6.4 × 10^−12^), and positive regulation of leukocyte activation (enrichment 32.6, *p* = 6.4 × 10^−12^). These findings suggest that reduced expression of these genes may impair the maturation and functional activity of immunocompetent cells in patients with tuberculosis (Figure 2B).

An enrichment analysis based on the KEGG database for downregulated genes revealed the highest enrichment for Th1/Th2 cell differentiation pathways (Th1 and Th2 cell differentiation, enrichment 134.8, *p* = 1.3 × 10^−14^) and Th17 cell differentiation (enrichment 130.4, *p* = 4.4 × 10^−16^), highlighting the role of these genes in immune regulation.

Of particular note was enrichment of the PD-L1 expression and PD-1 checkpoint pathway (enrichment 68.9, *p* = 2.4 × 10^−6^) and the T-cell receptor signaling pathway (enrichment 51.3, *p* = 7.1 × 10^−6^). This suggests that suppression of these genes may contribute to T-cell exhaustion characteristic of chronic infections. Pathways associated with viral infections (HTLV-1, hepatitis B, EBV, and HIV-1), cellular senescence (enrichment 49.4, *p* = 4.3 × 10^−7^), and oncogenesis (pathways in cancer, enrichment 29.3, *p* = 2.1 × 10^−12^), including acute and chronic myeloid leukemia, were also identified, which may reflect the involvement of these genes in fundamental processes of cell proliferation, differentiation, and survival (Figure 2B).

#### 3.2.3. Enrichment Analysis of Differentially Expressed Genes in COPD

Functional analysis of the upregulated DEGs identified in patients with COPD revealed their involvement in processes related to neutrophil activation, antimicrobial defense, and systemic inflammatory response.

**Figure 3:**
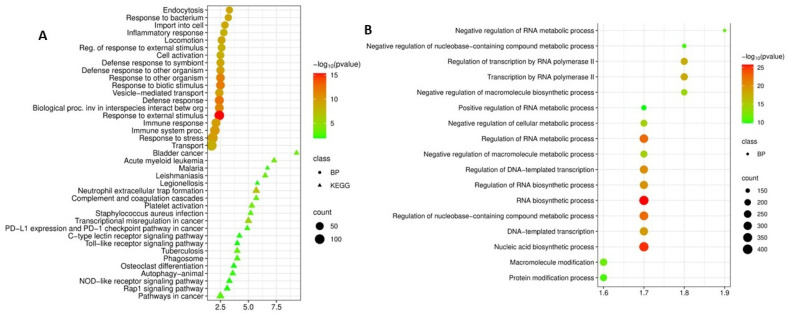
(**A**) Functional enrichment analysis of biological processes (BP) and KEGG pathways of upregulated differentially expressed genes in COPD versus control; (**B**) Functional enrichment analysis of biological processes and KEGG pathways of downregulated differentially expressed genes in COPD versus control.

Analysis of biological processes (Gene Ontology, Biological Process) revealed a significant enrichment of terms related to responses to external and biotic stimuli (Figure 3A). The most significant (by *p*-value) were processes related to response to external stimuli (*p* = 4.3 × 10^−16^), response to other organisms (*p* = 2.5 × 10^−12^), as well as the immune response in general (*p* = 4.8 × 10^−13^) and response to symbionts (*p* = 9.2 × 10^−10^). Another important finding was the high enrichment of biological processes associated with “response to bacteria” (fold enrichment 3.2, *p* = 9.2 × 10^−10^) and “defense response to symbiont” (fold enrichment 2.5, *p* = 9.2 × 10^−10^). This may reflect systemic activation of antimicrobial programs. The enrichment of processes associated with the “inflammatory response” (fold enrichment 2.8, *p* = 4.8 × 10^−9^), “cell activation” (fold enrichment 2.5, *p* = 3.4 × 10^−9^), and “immune response” (fold enrichment 2.1, *p* = 2.9 × 10^−10^) may confirm the generalized nature of immuno-inflammatory disorders in COPD. The presence in the list of enriched terms of the processes “endocytosis” (fold enrichment 3.3, *p* = 8.2 × 10^−10^) and “vesicle-mediated transport” (fold enrichment 2.4, *p* = 4.1 × 10^−10^) in the list of enriched terms may indicate the involvement of intracellular trafficking mechanisms, which may be associated with enhanced phagocytic activity of neutrophils and macrophages. Overall, GO analysis suggests that the set of genes with elevated expression in the blood in COPD may reflect a systemic inflammatory response characterized by the activation of the innate immune system, antimicrobial mechanisms, and cellular activation.

Analysis of pathway enrichment based on the KEGG database allowed us to identify the molecular mechanisms underlying the observed transcriptional changes (Figure 3A). The most significant finding was the detection of enrichment in the “Neutrophil extracellular trap formation (NETosis)” pathway (fold enrichment 5.7, *p* = 1.0 × 10^−7^). This is a central mechanism that plays a dual role in COPD: on the one hand, it ensures the elimination of pathogens, and on the other, when overactivated, it causes lung tissue damage and sustains chronic inflammation. The detection of NETosis in the blood may confirm the systemic nature of this process.

An important finding was the enrichment of key innate immune signaling pathways identified in the blood of COPD patients: the NOD-like receptor signaling pathway (fold enrichment 3.3, *p* = 9.5 × 10^−3^); the toll-like receptor signaling pathway (fold enrichment 4.0, *p* = 1.2 × 10^−2^); and the C-type lectin receptor signaling pathway (fold enrichment 4.2, *p* = 1.0 × 10^−2^). The simultaneous enrichment of these three sensory systems may serve as direct molecular evidence that fundamental mechanisms for recognizing pathogen-associated molecular patterns (PAMPs) and damage-associated molecular patterns (DAMPs) are chronically activated in COPD. Their systemic activation may be a key link in the maintenance of chronic inflammation and the development of comorbid conditions.

Enrichment of the “Phagosome” pathway (fold enrichment 4.0, *p* = 2.9 × 10^−3^) may indicate the activation of processes involved in the phagocytosis and degradation of microorganisms and cellular debris. At the same time, dysfunction of this pathway can lead to incomplete clearance of apoptotic cells and chronic inflammation. Enrichment of the “Complement and coagulation cascades” pathway (fold enrichment 5.7, *p* = 9.7 × 10^−4^) and “Platelet activation” (fold enrichment 5.3, *p* = 2.5 × 10^−4^) may reflect the association of systemic inflammation with the risk of thrombotic complications and cardiovascular diseases in COPD.

An enrichment of the “Tuberculosis” pathway (fold enrichment 4.0, *p* = 9.7 × 10^−4^) was also identified in the blood of patients with COPD. This result may indicate that transcriptional programs similar to those in tuberculosis are activated in COPD. Enrichment was also detected in pathways associated with intracellular infections: leishmaniasis (fold enrichment 6.5, *p* = 4.7 × 10^−4^), malaria (fold enrichment 6.7, *p* = 5.6 × 10^−3^), and legionellosis (fold enrichment 5.8, *p* = 9.4 × 10^−3^). When interpreting these results, it is important to note that the names of the identified pathways reflect database names but do not indicate a direct link to specific diseases. The enrichment of these pathways may indicate a general cellular response directed against intracellular pathogens.

Enrichment of pathways associated with oncological diseases, such as bladder cancer (fold enrichment 9.3, *p* = 4.7 × 10^−4^), acute myeloid leukemia (fold enrichment 7.3, *p* = 2.5 × 10^−4^), PD-L1 expression and PD-1 checkpoint (fold enrichment 4.9, *p* = 5.6 × 10^−3^), transcriptional misregulation in cancer (fold enrichment 5.0, *p* = 3.1 × 10^−6^), and the general “Cancer” pathway (Pathways in cancer, fold enrichment 2.5, *p* = 1.5 × 10^−3^), may reflect the activation of common signaling cascades associated with proliferation, inflammation, and suppression of apoptosis, which may create a favorable environment for oncogenesis.

Thus, analysis of upregulated DEGs in patients with COPD revealed a distinct and comprehensive systemic inflammatory signature. The data demonstrate not only the activation of neutrophil effector mechanisms (NETosis and phagocytosis), but also the involvement of fundamental innate immune pathways (TLR, NOD, CLR), as well as direct molecular parallels with tuberculosis. These results may serve as confirmation that COPD is a systemic disease and that the chronic inflammation associated with it is persistent and generalized in nature.

Functional analysis of the downregulated DEGs in patients with COPD revealed their broad involvement in the regulation of transcription and RNA metabolism (Figure 3B). Biological process analysis (Gene Ontology, Biological Process) demonstrated that genes with downregulated expression were significantly enriched in processes related to transcription mediated by RNA polymerase II (fold enrichment 1.8, *p* = 8.6 × 10^−19^) and regulation of transcription by RNA polymerase II (fold enrichment 1.8, *p* = 1.7 × 10^−18^). The most statistically significant enrichment was observed for nucleic acid biosynthetic processes (fold enrichment 1.7, *p* = 7.3 × 10^−26^) and DNA-templated transcription (fold enrichment 1.7, *p* = 5.6 × 10^−20^). These data may indicate a global suppression of transcriptional activity in the cells of the peripheral blood of patients with COPD. Significant enrichment was found for the processes of negative regulation of RNA metabolism (fold enrichment 1.9, *p* = 5.1 × 10^−12^) and negative regulation of metabolism of nucleobase-containing compounds (negative regulation of nucleobase-containing compound metabolic process, fold enrichment 1.8, *p* = 4.8 × 10^−11^). This may indicate a systemic suppression of RNA synthesis and processing, which may reflect depletion of cellular resources and disruption of adaptive mechanisms.

Enrichment of DNA-templated transcription (fold enrichment 1.7, *p* = 5.6 × 10^−20^) and nucleic acid biosynthetic processes (fold enrichment 1.7, *p* = 7.3 × 10^−26^) may confirm the fundamental nature of transcriptional disturbances in COPD. Among the enriched processes, positive regulation of RNA metabolic processes (fold enrichment 1.7, *p* = 1.6 × 10^−10^) was also identified, which may indicate the complex nature of regulatory changes involving both activating and repressing mechanisms of gene expression control.

Taken together, the data obtained may indicate that in COPD there is a global suppression of transcriptional activity in peripheral blood, affecting a wide range of genes involved in the regulation of RNA synthesis. This may be related to exhaustion of immunocompetent cells, disruption of their functional activity, and the development of the phenomenon of “immune exhaustion” characteristic of chronic inflammatory diseases. The observed changes overlap with the results obtained in the analysis of tuberculosis, where suppression of key transcriptional regulators was also observed, which suggests the presence of common mechanisms of systemic immunosuppression in both diseases.

#### 3.2.4. Functional Analysis of Key Hub Genes in COPD

For the validation of the results of whole-genome analysis and identification of central regulators of the pathological process in COPD, enrichment analysis was carried out for the 15 most significant upregulated genes identified using Cytoscape (*OASL*, *HELZ2*, *IFIT2*, *CTSG*, *IFI35*, *GBP1*, *STAT1*, *IFIT3*, *PARP14*, *PARP9*, *ELANE*, *IRF7*, *IFIT1*, *ISG15*, *IFI6*).

An analysis of biological processes (GO BP) for this set of genes revealed their central role in the implementation of antiviral mechanisms of the innate immune system. The highest enrichment scores were observed for the interferon-mediated signaling pathway (enrichment 66.3, *p* = 3.6 × 10^−7^), defense response to virus (enrichment 40.7, *p* = 9.3 × 10^−13^), and the response to viruses (enrichment 32.2, *p* = 3.7 × 10^−12^). These data highlight the key role of antiviral immunity in the pathogenesis of COPD, which is consistent with current understanding of viral infections as an important trigger for disease exacerbations.

**Figure 4:**
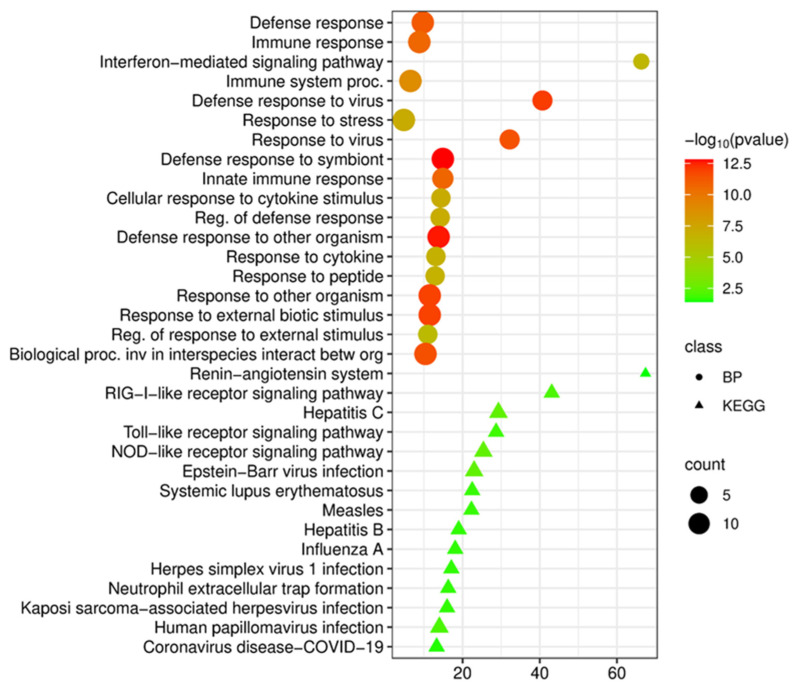
Functional enrichment analysis of biological processes (BP) and KEGG pathways of the top 15 most significant upregulated differentially expressed genes in COPD versus control.

Significant enrichment was also detected for the defense response to symbionts (defense response to symbiont, enrichment 14.9, *p* = 1.5 × 10^−13^), the innate immune response (innate immune response, enrichment 14.9, *p* = 1.8 × 10^−11^), and the defense response to other organisms (enrichment 13.8, *p* = 2.2 × 10^−13^). In addition, enrichment was observed for cellular response to cytokine stimulus (enrichment 14.4, *p* = 7.7 × 10^−8^) and regulation of defense response (enrichment 14.2, *p* = 8.4 × 10^−8^) (Figure 4).

A natural result was the enrichment of more general processes reflecting the systemic nature of the immune response in COPD. A significant enrichment was observed for terms related to response to other organisms (Response to other organism, enrichment 11.5, *p* = 1.2 × 10^−12^), response to external biotic stimulus (Response to external biotic stimulus, enrichment 11.5, *p* = 1.2 × 10^−12^), response to cytokines (Response to cytokine, enrichment 13.1, *p* = 1.6 × 10^−7^), and response to peptides (Response to peptide, enrichment 12.9, *p* = 1.7 × 10^−7^). Taken together, the data indicate that the identified hub genes coordinate key mechanisms of innate antiviral immunity, which in COPD may reflect systemic activation of the immune system (Figure 4).

An enrichment analysis based on the KEGG database revealed significant enrichment of signaling pathways associated with infectious diseases and the regulation of the inflammatory response (Figure 4). The toll-like receptor signaling pathway (enrichment 28.7, *p* = 1.4 × 10^−2^) and NOD-like receptor signaling pathways (enrichment 25.4, *p* = 3.6 × 10^−3^) were identified, confirming the key role of innate immune receptors in the recognition of pathogen-associated molecular patterns in COPD. An enrichment of the neutrophil extracellular trap (NET) formation pathway was also identified (enrichment 16.3, *p* = 2.1 × 10^−2^), which is consistent with the known role of NETosis in lung tissue damage in COPD.

The highest enrichment was observed for the renin-angiotensin system (enrichment 67.4, *p* = 3.9 × 10^−2^), which is of interest in the context of the regulation of vascular tone, fibrosis, and inflammation in chronic lung diseases. High enrichment was also detected for the RIG-I-like receptor signaling pathway (enrichment 43.1, *p* = 9.2 × 10^−3^), which plays a key role in the recognition of viral RNA.

#### 3.2.5. Functional Analysis of Common Upregulated Differentially Expressed Genes in COPD and Tuberculosis

**Figure 5:**
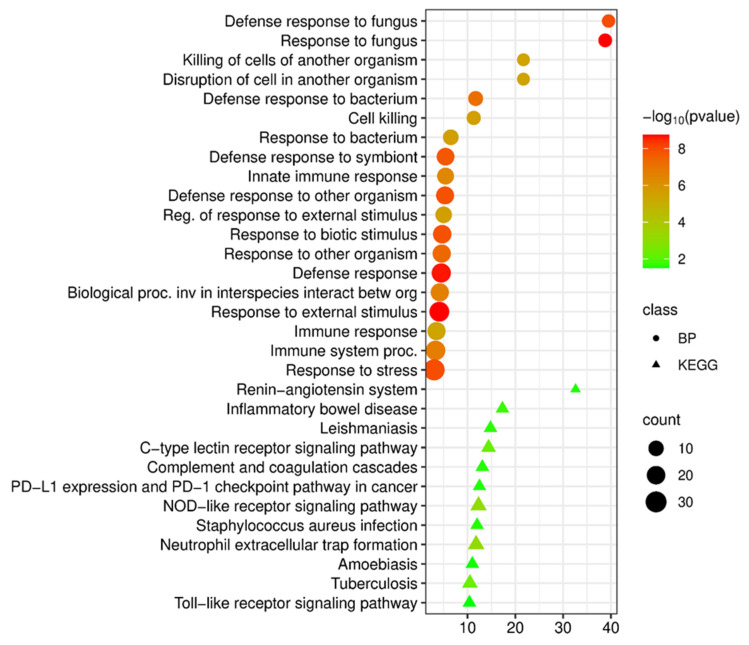
Functional enrichment analysis of biological processes (BP) and KEGG pathways of common upregulated differentially expressed genes in tuberculosis and COPD.

An analysis conducted using the FunRich tool (v. 3.1.3) identified 66 upregulated genes common to COPD and tuberculosis. Biological process analysis (Gene Ontology, Biological Process) revealed a key role of the shared upregulated genes in the regulation of inflammation (Figure 5).

Analysis of biological processes (GO Biological Process) revealed a significant enrichment of terms related to the defense response and the response to external stimuli (Figure 5). The highest fold enrichment values were observed for processes associated with the antifungal response: defense against fungi (fold enrichment 39.5, *p* = 1.1 × 10^−8^) and response to fungi (fold enrichment 38.8, *p* = 1.8 × 10^−9^). Enrichment was detected for processes of killing cells of another organism (fold enrichment 21.7, *p* = 3.7 × 10^−6^) and cell death (fold enrichment 11.3, *p* = 2.5 × 10^−6^), which may reflect the activation of cytotoxic mechanisms.

Significant enrichment was observed for processes associated with the bacterial response: defense against bacteria (fold enrichment 11.7, *p* = 7.0 × 10^−8^) and response to bacteria (fold enrichment 6.5, *p* = 2.7 × 10^−6^). Enrichment was also identified for processes of the innate immune response (fold enrichment 5.4, *p* = 3.8 × 10^−7^), the protective response to a symbiont (fold enrichment 5.4, *p* = 1.9 × 10^−8^), and the defensive response to other organisms (fold enrichment 5.3, *p* = 1.4 × 10^−8^). A significant enrichment of the inflammatory immune response itself (fold enrichment 3.5, *p* = 3.7 × 10^−6^) and the immune system as a whole (fold enrichment 3.3, *p* = 1.7 × 10^−7^) was also detected, which may reflect the central role of chronic inflammation in the pathogenesis of both diseases (Figure 5).

An analysis of signal pathway enrichment based on the KEGG database demonstrated that the common genes are involved in a wide range of immune and infectious signaling cascades (Figure 5). The highest fold enrichment scores were observed for the renin-angiotensin system (fold enrichment 32.6, *p* = 2.5 × 10^−2^), which is of particular interest in the context of the regulation of vascular tone, fibrosis, and inflammation in chronic lung diseases.

At the molecular level, the common genes were significantly associated with key signaling pathways of the innate immune system, including NOD-like receptors (fold enrichment 12.3, *p* = 7.2 × 10^−4^), C-type lectin receptors (fold enrichment 14.4, *p* = 5.4 × 10^−3^), and toll-like receptors (fold enrichment 10.4, *p* = 3.1 × 10^−2^). Enrichment was also observed for neutrophil extracellular trap (NET) formation (fold enrichment 11.8, *p* = 7.2 × 10^−4^) and complement and coagulation cascades (fold enrichment 13.1, *p* = 2.5 × 10^−2^), as well as PD-L1 expression and the PD-1 checkpoint pathway (fold enrichment 12.5, *p* = 2.5 × 10^−2^). Significant enrichment was identified for infectious disease pathways, which may reflect common mechanisms of the immune response to various classes of pathogens: leishmaniasis (fold enrichment 14.8, *p* = 2.3 × 10^−2^), inflammatory bowel disease (fold enrichment 17.3, *p* = 1.8 × 10^−2^), amebiasis (fold enrichment 11.0, *p* = 2.9 × 10^−2^), and *Staphylococcus aureus* infection (fold enrichment 12.0, *p* = 2.5 × 10^−2^). Of particular note is the significant enrichment of the tuberculosis signaling pathway (fold enrichment 10.5, *p* = 4.8 × 10^−3^), which may confirm the relevance of the identified common genes for the diseases under study (Figure 5).

#### 3.2.6. Functional Analysis of Common Most Significant Upregulated Differentially Expressed Genes in COPD and Tuberculosis

To identify central regulatory molecules that determine the commonality of pathogenetic mechanisms of COPD and tuberculosis, functional enrichment analysis was carried out for the 15 most significant upregulated genes shared by both diseases (*MS4A3*, *DEFA4*, *RNASE2*, *CTSG*, *S100A12*, *SLPI*, *STAT1*, *IFIT1*, *ARG1*, *ELANE*, *GBP1*, *TLR4*, *CAMP*, *CEACAM6*, *MPO*).

**Figure 6:**
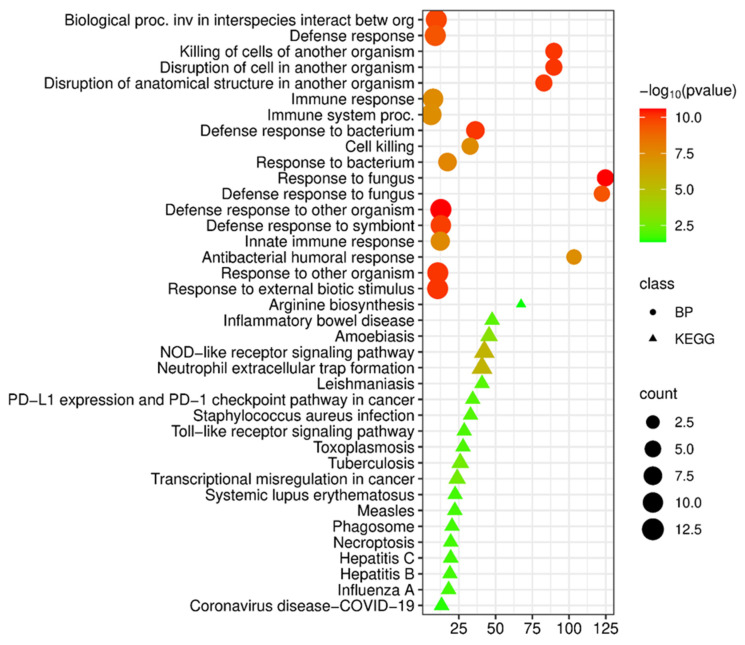
Functional enrichment analysis of biological processes (BP) and KEGG pathways of shared key upregulated differentially expressed genes in tuberculosis and COPD.

Biological process analysis for this set of genes demonstrated their role in the effector mechanisms of innate immunity, with a strong emphasis on antimicrobial defense and direct elimination of pathogens (Figure 6). The highest enrichment values were related to the antifungal response: “response to fungus” (fold enrichment 124.8, *p* = 2.6 × 10^−11^) and “defense response to fungus” (fold enrichment 122.4, *p* = 4.0 × 10^−10^). This indicates that the identified hub genes encode proteins critically important for the recognition and elimination of fungal pathogens.

High enrichment values were also found for processes related to the direct killing of cells of other organisms: “killing of cells of another organism” (fold enrichment 89.7, *p* = 8.5 × 10^−11^), “disruption of cell in another organism” (fold enrichment 89.7, *p* = 8.5 × 10^−11^), and “disruption of anatomical structure in another organism” (fold enrichment 82.9, *p* = 1.0 × 10^−10^). These data may confirm that the shared hub genes are key effector molecules that provide killing of pathogens.

An important result was the high enrichment of “antibacterial humoral response” (fold enrichment 103.4, *p* = 4.4 × 10^−8^) and of processes related to antibacterial defense: “defense response to bacterium” (fold enrichment 36.3, *p* = 8.5 × 10^−11^) and “response to bacterium” (fold enrichment 17.3, *p* = 1.9 × 10^−8^). This may confirm the key role of these genes in fighting bacterial infections, which is especially relevant both for tuberculosis and for bacterial exacerbations in COPD.

Enrichment of the processes of “cell killing” (fold enrichment 32.7, *p* = 3.7 × 10^−8^) and “innate immune response” (fold enrichment 12.4, *p* = 2.8 × 10^−8^) complements the overall picture. Taken together, the data obtained may indicate that the shared hub genes function as key effectors of innate immunity that provide recognition and direct killing of a wide range of pathogens (bacteria and fungi).

KEGG enrichment analysis made it possible to identify specific signaling pathways in which the shared hub genes are involved and confirmed the results of GO analysis. The highest enrichment values were obtained for the NOD-like receptor signaling pathway (fold enrichment 42.4, *p* = 3.0 × 10^−6^). NLR receptors are key sensors of intracellular pathogens and components of inflammasomes, which emphasizes the role of shared genes in triggering the inflammatory response.

An important result was the high enrichment of the “Neutrophil extracellular trap formation (NETosis)” pathway (fold enrichment 40.8, *p* = 3.0 × 10^−6^). This mechanism is one of the main effector pathways of neutrophils, leading to the release of degranulating enzymes and DNA nets that provide capture and killing of pathogens. Significant enrichment was also detected for the toll-like receptor signaling pathway (fold enrichment 28.7, *p* = 1.3 × 10^−2^). TLR4 is a key component of this pathway, which provides recognition of both mycobacterial antigens (lipopolysaccharide) and damage-associated molecular patterns (DAMPs) released during smoking and destruction of lung tissue.

An important result was the direct enrichment of the tuberculosis pathway (fold enrichment 26.0, *p* = 3.0 × 10^−3^), which may confirm the relevance of the identified genes for the disease under study, as well as the enrichment of the pathways of phagocytosis (phagosome, fold enrichment 20.4, *p* = 1.8 × 10^−2^) and necroptosis (fold enrichment 19.5, *p* = 1.8 × 10^−2^). The presence in the list of enriched pathways of “Inflammatory bowel disease” (fold enrichment 47.7, *p* = 8.2 × 10^−3^) and “Systemic lupus erythematosus” (fold enrichment 22.5, *p* = 1.7 × 10^−2^) may indicate the commonality of immune-inflammatory mechanisms in various chronic inflammatory diseases.

Thus, the analysis of 15 key hub genes made it possible to identify them as central effectors and regulators of innate immunity. High enrichment values for the processes of direct killing of pathogens, NETosis, and NOD signaling may emphasize their critical role in the pathogenesis of both COPD and tuberculosis.

## 4. Discussion

In the present study, a comprehensive bioinformatic analysis of the transcriptomic profiles of patients with pulmonary tuberculosis and COPD was carried out in order to identify shared pathogenetic mechanisms. Despite the different etiologies of these diseases, the results obtained suggest the potential presence of common molecular pathways and key regulatory genes determining the similarity of immunopathological processes in these diseases [19].

The most significant result of our study was the identification of DEGs shared between COPD and tuberculosis that are involved in the key effector mechanisms of innate immunity. Enrichment analysis for the 15 shared upregulated hub genes (MS4A3, DEFA4, RNASE2, CTSG, S100A12, SLPI, STAT1, IFIT1, ARG1, ELANE, GBP1, TLR4, CAMP, CEACAM6, MPO) demonstrated high enrichment values for processes related to direct killing of pathogens, including “killing of cells of another organism”, “neutrophil extracellular trap formation (NETosis)”, and “antibacterial humoral response”.

Neutrophil extracellular trap formation (NETosis) is one of the main effector pathways of neutrophils, leading to the release of degranulating enzymes (elastase, cathepsin G, myeloperoxidase) and DNA nets that provide capture and killing of pathogens. In COPD, NETosis makes a dual contribution: on the one hand, it ensures the elimination of bacteria colonizing the airways, and on the other, upon excessive activation, it causes damage to lung tissue and supports chronic inflammation [37,38]. In tuberculosis, NETosis also plays a protective role; however, its hyperactivation may contribute to the destruction of the lung parenchyma [39]. The identification of NETosis in the analysis of the peripheral blood of patients with COPD may confirm the systemic nature of this process, which is consistent with current views on COPD as a systemic disease [40,41]. Notably, longitudinal whole-blood transcriptomic profiling demonstrated co-induction of neutrophil degranulation and NETosis pathway genes up to six months prior to tuberculosis diagnosis, and these signatures were further enriched in patients with more extensive lung damage on PET-CT, underscoring the systemic footprint of neutrophil-mediated defense in TB [42].

The NOD-like receptor signaling pathway (in the analysis of 15 hub genes) is also of interest. NLR receptors are key sensors of intracellular pathogens and components of inflammasomes, which emphasizes the role of the shared genes in triggering the inflammatory response. In tuberculosis, activation of the NLRP3 inflammasome is a critical link in the recognition of mycobacterial antigens and the production of IL-1β and IL-18 [43]. In COPD, DAMPs (damage-associated molecular patterns), released during smoking and destruction of lung tissue, also activate NOD-like receptors, supporting chronic inflammation [44]. The detection of enrichment of this pathway in the blood of patients with COPD may be considered confirmation of systemic activation of innate immunity.

The toll-like receptor signaling pathway and, in particular, TLR4, which was included in the list of top 15 hub genes, provides recognition of both the lipopolysaccharide of mycobacteria and DAMPs released during cell damage. In tuberculosis, TLR2/4/9 recognize lipoproteins and DNA of mycobacteria [45], and in COPD, TLR4 is activated by DAMPs (HMGB1, HSP, and fragments of the extracellular matrix) released during smoking [44]. These results may confirm that the shared hub genes are key sensors integrating signals from both infectious and noninfectious stimuli.

Activation of TLR and NOD signaling pathways is closely linked to the macrophage phenotype. In tuberculosis, M1 polarization is essential for controlling the infection, whereas a shift toward the M2 phenotype contributes to chronicity and fibrosis. In COPD, by contrast, smoking induces a mixed M1/M2 phenotype with a predominance of pro-inflammatory M1 macrophages, which exacerbates lung tissue destruction, while ineffective phagocytosis is associated with impaired macrophage phenotype switching [46,47,48]. The identification of ARG1 (M2 marker) and STAT1 (M1 signaling mediator) among common key genes confirms the involvement of macrophage polarization in the common mechanisms underlying COPD and tuberculosis.

Another important common link identified in our analysis is the phagosome pathway, which was significantly enriched in both COPD and tuberculosis, particularly among the common key genes. The functional activity of phagosomes is crucial not only for the elimination of pathogens but also for the “clearance” of cellular debris. In tuberculosis, *Mycobacterium tuberculosis* uses phagosomes as a niche for survival, disrupting their maturation and fusion with lysosomes [49]. In COPD, by contrast, the central problem is not so much infection as a disruption of the efferocytosis process—the removal of apoptotic neutrophils and other dead cells by alveolar macrophages. Dysfunction of the phagosome apparatus in COPD leads to the accumulation of cellular debris, which sustains chronic sterile inflammation and autoimmune reactions [50,51]. Thus, the enrichment of the phagosome pathway indicates that in both diseases there is a profound reorganization of intracellular clearance processes, but with different functional consequences: in tuberculosis, this contributes to pathogen persistence, while in COPD, it leads to chronic inflammation and damage to lung tissue.

Analysis of the hub upregulated common DEGs revealed a significant enrichment of processes associated with the antifungal response: the antifungal defense response and the response to fungi. This may indicate that common transcriptional programs aimed at eliminating fungal pathogens are activated in COPD and tuberculosis. Given that fungal colonization of the airways is common in COPD and is also possible in immunocompromised patients with tuberculosis, the identified enrichment may reflect systemic activation of antifungal immunity.

Enrichment of the renin-angiotensin system pathway is of particular interest in the context of the regulation of vascular tone, fibrosis, and inflammation in chronic lung diseases. It is known that the local renin-angiotensin system plays an important role in the remodeling of the airways and lung tissue in COPD and may also modulate the inflammatory response in tuberculosis [52,53]. Identification of activation of this pathway in patients with both diseases may indicate common mechanisms underlying vascular and fibrotic complications or may be associated with comorbid conditions.

Significant enrichment was identified for pathways related to infectious diseases, reflecting common mechanisms of the immune response to various classes of pathogens: leishmaniasis, inflammatory bowel disease, amebiasis, and *Staphylococcus aureus* infection. When interpreting these results, it is important to note that the names of the identified pathways reflect database names but do not indicate a direct link to specific diseases. The enrichment of these pathways may indicate a general cellular response directed against intracellular pathogens and is consistent with current understanding of the role of bacterial infections in COPD exacerbations and the pathogenesis of tuberculosis.

The enrichment of the PD-L1 expression and PD-1 checkpoint pathway among the overall upregulated genes is of interest. The PD-1/PD-L1 signaling pathway is a key regulator of T-cell exhaustion in chronic infections, including tuberculosis, and may also play a role in the immunopathogenesis of COPD. The identification of enrichment of this pathway in patients with both diseases may indicate the presence of common mechanisms of immunosuppression and T-cell dysfunction.

Interestingly, in COPD we also observed global suppression of transcriptional activity in peripheral blood (enrichment of the processes of negative regulation of RNA metabolism). This may indicate the presence of systemic immunosuppression or “immune exhaustion” in COPD, which is a new and potentially important finding that requires further study. These observations fit with growing evidence that immune checkpoint pathways are dysregulated in COPD and that lung T cells in COPD patients upregulate PD-1 and lose cytotoxic function, consistent with a chronic-infection-like exhaustion phenotype [54,55].

In COPD, KEGG pathway analysis of the top 15 upregulated genes revealed a mixed signature involving both antiviral and neutrophilic inflammatory pathways. Significant enrichment was observed for the toll-like receptor signaling pathway and NOD-like receptor signaling pathway, confirming the key role of innate immune receptors in COPD pathogenesis. Notably, enrichment was also detected for the neutrophil extracellular trap formation (NETosis) pathway, which is one of the most significant mechanisms of tissue damage in chronic lung inflammation. These data may emphasize the key role of neutrophils in the pathogenesis of COPD, which is consistent with numerous studies demonstrating neutrophilic inflammation as one of the central characteristics of the disease [37,56]. Direct enrichment of the tuberculosis pathway may confirm the relevance of the identified shared genes to the disease under study and serves as an internal validation of our approach.

Our results are consistent with several recent studies. First, the role of neutrophil-mediated effector mechanisms—including NETosis, degranulation, and cytotoxicity—as a convergent feature of both COPD and tuberculosis has been highlighted in recent comparative reviews of neutrophils in infectious and non-infectious pulmonary disorders [56]. Our data confirm this at the transcriptional level, showing that the neutrophilic signature is shared by COPD and tuberculosis.

Second, systematic review evidence indicates that COPD is independently associated with an increased risk of developing active tuberculosis [19]; our study complements these data by showing that not only epidemiological risk but also convergent changes in the expression of innate-immunity genes (for example, TLR4, MPO, and ELANE) may underlie the commonality of pathogenesis.

Third, our observation of suppression of transcriptional activity in COPD is consistent with a growing number of studies indicating the presence of immune aging and T cell exhaustion in COPD. The work of McKendry et al. demonstrated increased expression of PD-1 on CD8^+^ T cells of patients with COPD together with loss of cytotoxic function [55], and this observation has been integrated into the broader framework of immune checkpoint dysregulation in COPD [54]; these findings overlap with our observation of enrichment of the PD-L1 pathway among the downregulated genes in tuberculosis and raise the question of whether a similar mechanism may operate in COPD.

The present study has a number of limitations that should be taken into account when interpreting the results. First, the small sample size for tuberculosis: the GSE34608 dataset included only 8 patients with tuberculosis. Despite the use of strict statistical criteria (FDR ≤ 5%, |logFC| > 1), the small sample size increases the risk of false-positive and false-negative results. The results require validation in larger independent cohorts. Second, transcriptomic changes do not always correlate with the levels of the corresponding proteins. Further studies at the protein level are required to confirm the functional significance of the identified genes. Third, we did not have access to individual clinical data of patients (age, sex, treatment, disease stage, presence of complications, or comorbidities), which does not allow us to assess the influence of these factors on gene expression and to perform a stratified analysis. Fourth, although the use of whole blood for both diseases is a methodological advantage, we cannot completely exclude the influence of blood cell composition on the identified transcriptional changes. In addition, we used different cutoff criteria for the identification of DEGs in the COPD and tuberculosis datasets, which was due to the need to ensure a balance between statistical power and biological interpretability. It is important to note that our study is hypothesis-generating. The results obtained require experimental validation using functional methods and clinical verification in independent prospective cohorts.

## 5. Conclusions

As a result of the bioinformatic analysis carried out, shared molecular signatures and key regulatory genes were identified that may determine the similarity of the pathogenetic mechanisms of chronic obstructive pulmonary disease and pulmonary tuberculosis. The key shared links are the activation of innate immunity with a central role of neutrophils, the formation of neutrophil extracellular traps (NETosis), and the activation of NOD-like and toll-like receptors. At the molecular level, the shared mechanisms include those related to the production of nitric oxide, proinflammatory cytokines, and activation of the transcription factor NF-κB. These data may support the hypothesis that COPD and tuberculosis, despite their different etiologies, have some similar effector mechanisms.

The genes and signaling pathways we have identified may serve as candidates for the development of biomarkers (in particular, NETosis markers for assessing the risk of progression and lung tissue destruction) and as potential therapeutic targets. However, all these findings require further validation in experimental and clinical studies. The data obtained also open up prospects for developing approaches to predict the risk of tuberculosis development in patients with COPD based on the analysis of key gene expression, which must also be confirmed in independent cohorts. Further experimental and clinical studies are necessary to validate the results obtained and assess their translational potential.

## Data Availability

The original data presented in the study are openly available in GEO, NCBI. The raw data supporting the conclusions of this article will be made available by the corresponding authors on request.
